# Alterations in the CD56^−^ and CD56^+^ T Cell Subsets during COVID-19

**DOI:** 10.3390/ijms24109047

**Published:** 2023-05-20

**Authors:** Julia D. Vavilova, Maria O. Ustiuzhanina, Anna A. Boyko, Maria A. Streltsova, Sofya A. Kust, Leonid M. Kanevskiy, Rustam N. Iskhakov, Alexander M. Sapozhnikov, Ekaterina O. Gubernatorova, Marina S. Drutskaya, Mikhail V. Bychinin, Oksana N. Novikova, Anna G. Sotnikova, Gaukhar M. Yusubalieva, Vladimir P. Baklaushev, Elena I. Kovalenko

**Affiliations:** 1 Shemyakin & Ovchinnikov Institute of Bioorganic Chemistry, Russian Academy of Sciences, 117997 Moscow, Russia; juliateterina12@gmail.com (J.D.V.); sonya.erokhina@gmail.com (S.A.K.); amsap@mail.ru (A.M.S.); 2 Center of Life Sciences, Skolkovo Institute of Science and Technology, 121205 Moscow, Russia; 3 Center for Precision Genome Editing and Genetic Technologies for Biomedicine, Engelhardt Institute of Molecular Biology, Russian Academy of Sciences, 119991 Moscow, Russia; 4 Division of Immunobiology and Biomedicine, Sirius University of Science and Technology, Sirius, Krasnodarsky Krai, 354349 Sochi, Russia; 5 Federal Research and Clinical Center of Specialized Medical Care and Medical Technologies FMBA of Russia, 115682 Moscow, Russia; 6 Engelhardt Institute of Molecular Biology, Russian Academy of Sciences, 119991 Moscow, Russia

**Keywords:** COVID-19, NKT-like cells, T cells, CD56^+^ T cells, PD1, HLA-DR, KIR2DL2/3, NKp30

## Abstract

The effectiveness of the antiviral immune response largely depends on the activation of cytotoxic T cells. The heterogeneous group of functionally active T cells expressing the CD56 molecule (NKT-like cells), that combines the properties of T lymphocytes and NK cells, is poorly studied in COVID-19. This work aimed to analyze the activation and differentiation of both circulating NKT-like cells and CD56^−^ T cells during COVID-19 among intensive care unit (ICU) patients, moderate severity (MS) patients, and convalescents. A decreased proportion of CD56^+^ T cells was found in ICU patients with fatal outcome. Severe COVID-19 was accompanied by a decrease in the proportion of CD8^+^ T cells, mainly due to the CD56^−^ cell death, and a redistribution of the NKT-like cell subset composition with a predominance of more differentiated cytotoxic CD8^+^ T cells. The differentiation process was accompanied by an increase in the proportions of KIR2DL2/3^+^ and NKp30^+^ cells in the CD56^+^ T cell subset of COVID-19 patients and convalescents. Decreased percentages of NKG2D^+^ and NKG2A^+^ cells and increased PD-1 and HLA-DR expression levels were found in both CD56^−^ and CD56^+^ T cells, and can be considered as indicators of COVID-19 progression. In the CD56^−^ T cell fraction, increased CD16 levels were observed in MS patients and in ICU patients with lethal outcome, suggesting a negative role for CD56^−^CD16^+^ T cells in COVID-19. Overall, our findings suggest an antiviral role of CD56^+^ T cells in COVID-19.

## 1. Introduction

Coronavirus Disease 2019 (COVID-19) caused by severe acute respiratory syndrome coronavirus-2 (SARS-CoV-2) leads to complex immune dysregulation and affects different components of the antiviral cellular immunity. In particular, T cell immune response, which plays an essential role in infection control, may vary in patients with mild, moderate, and severe COVID-19 [1,2]. The question about the predictive significance of different T cell markers of activation, differentiation, or exhaustion in COVID-19 remains relevant.

The specific adaptive response, induced by the recognition of a certain viral peptide presented by MHC molecules, is a hallmark of T cells. CD56^+^ T cells, the so-called NKT-like cells, constitute a subset of T cells that express both T cell receptor (TCR) and NK cell receptors, such as killer-cell immunoglobulin-like receptors (KIRs), Fc receptor FcγRIII (CD16), C-type lectin-like receptors (NKG2D, NKG2C, etc.), and others. Thus, NKT-like cells exhibit both adaptive and innate cell properties [3]. This subset includes alpha beta (αβ) T cells, most of which are CD8^+^ cells at the late stages of differentiation, gamma delta (γδ) T cells, and mucosa-associated invariant T (MAIT) cells [4], all of them characterized by increased effector functions including NK-cell-like cytotoxic potential [5,6]. It has been proposed that CD56 surface expression in CD8^+^ T cells is closely correlated with their cytotoxic activity [7]. Changes in the number, phenotype, and function of the CD56^+^ T cell fraction and their immunostimulatory effector role were shown in patients infected with such viruses as HIV and hepatitis C [5,8].

Over the past two years, most reports on COVID-19 have been highlighting lymphopenia as the main feature reflecting the severity of COVID-19 [9,10]. T cell subsets undergo qualitative changes in patients with COVID-19, i.e., through the reduction in the CD8^+^ subset [11,12,13,14,15], likely caused by COVID-19-induced cell death [16]. It has been shown earlier that CD8^+^ cells constitute the majority of the NKT-like subset [17]. Several publications emphasize an increase in CD8^+^ T cells with more mature phenotype during SARS-CoV-2 infection [14,18,19,20].

The reduction in the CD3^+^CD56^+^ cell fraction during COVID-19 in different cohorts of patients was observed in several studies [2,13,21], although other studies failed to confirm such trends [2,22,23]. The percentage of NKT-like cells is suggested to be a predictive biomarker for COVID-19 severity and patient outcome [2,21], although in another study alterations in circulating CD56^+^ T cell frequency were shown to be independent of COVID-19 severity [22,23]. Thus, the data on the size of NKT-like cell fraction in COVID-19 are inconsistent, and the use of CD56^+^ T cell subset size as a biomarker of COVID-19 severity needs more detailed consideration.

There is some evidence of T cell hyperactivation during COVID-19, which is associated with a poor clinical outcome of this disease [12]. Several studies have reported an activated phenotype of CD8^+^ T cells in patients with severe COVID-19, characterized by an increased expression of CD38 or/and HLA-DR molecules [11,24,25,26]. The T cell exhaustion can be considered as an additional indicator of COVID-19 progression [21]. Increased surface levels of the exhaustion markers such as PD-1, CTLA-4, and TIGIT on the peripheral blood T cells during severe COVID-19 were reported by several research groups [27,28]. In some cases, such an increase was associated with a reduced level of functional molecules, including IFN-γ, TNF-α, and IL-2, and could be used to predict the disease progression in COVID-19 patients [27].

CD56^+^ T cells often express KIRs, which interact with HLA class I molecules to modulate NK cell functional response [29]. KIR^+^ T lymphocytes are mainly CD8^+^CD27^−^CD28^−^ and express CD45RA and CD57 but not CCR7; thus, these are effector memory T cells re-expressing CD45RA (TEMRA) [29]. Elevated levels of KIR^+^CD8^+^ T cells in SARS-CoV-2 or influenza-infected patients correlated with the disease severity and vasculitis, however the authors strongly supported the hypothesis that KIR^+^CD8^+^ T cells perform a regulatory role in infection diseases, including COVID-19, by controlling autoreactive T cell response [30]. The frequency of a KIR^+^CD56^+^ T cell subset can increase dramatically in cytomegalovirus (HCMV) infection [31], but it is still unknown how this subpopulation responds to SARS-CoV-2. It was reported in 2020 that COVID-19 progression is accompanied by an increase in NKG2A expression in T cells, whereas a considerable reduction in NKG2A level is typical for convalescents [27,32]. Unlike inhibitory receptors present on NKT-like cells, which recognize MHC-I/MHC-like molecules, activating receptors (such as NKp30 and NKG2D) recognize ligands on the altered cells (tumor cells, virus-infected cells). It is known that a minor subpopulation of CD8^+^ T cells expressing NKp30-activating receptor exhibits a high potential for natural killer cell activity against tumor formations, and that IL-15 is able to induce the expression of NKp30 in a population of CD8^+^ T cells de novo [33]. NK-receptor-expressing CD8^+^ T cells are often characterized by an effector memory phenotype enriched within virus-specific T cells [34]. In contrast, the NKp30^+^CD8^+^ T cell population in the peripheral blood was shown to be CCR7^+^CD45RA^+^CD28^+^, indicating a naïve phenotype [33]. According to the previously published data, cytotoxic T cells co-expressing CD56^+^ are negative for NKp30 [33]. The role of CD56^+^ T cells in COVID-19 has been poorly described.

The prediction of further challenges associated with the emergence of new strains of SARS-CoV-2 is problematic. The Wuhan variant [35], which dominated the early stages of the pandemic, caused more severe cases of COVID-19 and high mortality. In this work, we focused on the phenotype analysis of the circulating T lymphocytes from the patients with COVID-19 collected in May–July 2020 in Moscow during the first wave of the pandemic. This research focused on the investigation of the CD56^−^ and CD56^+^ T cell subsets in the context of their differentiation and activation, including both classical markers of T cell differentiation and expression of activating and inhibitory NK cell receptors in samples from severe and moderate COVID-19 patients (32 and 28, respectively). Additionally, we examined changes in the most cytotoxic CD8^+^CD56^+^ subset level and granzyme B expression in T cells against the background of COVID-19. Further, we compared these features between T cells from the survivors and non-survivors in the group of patients with severe COVID-19.

## 2. Results

### 2.1. The Severe Cases of COVID-19 Are Accompanied by a Decrease in the Proportion of CD8^+^ T Cells, Mainly CD56^−^, and Changes in the CD56^+^ Fraction towards a Predominance of More Differentiated Cytotoxic Cells

**Figure 1:**
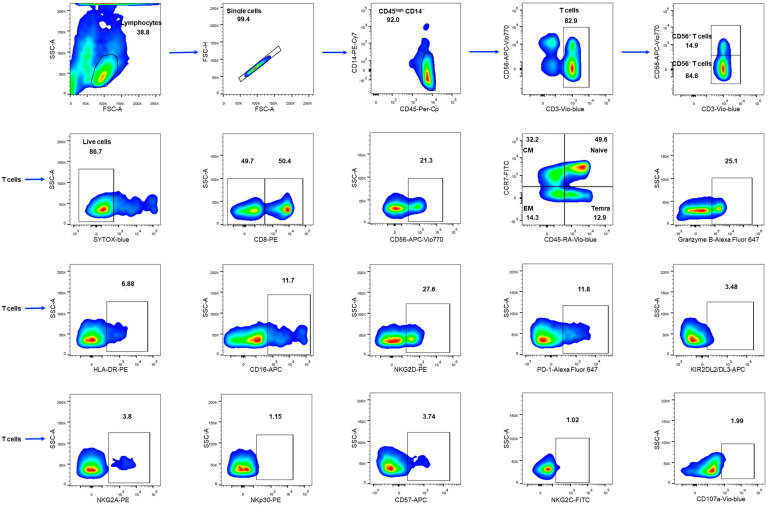
The gating strategy for cytometric analysis of T cells in PBMC samples. Threshold was set to cut off events with low CD45 staining. T cells were defined as CD3^+^ cells in the CD45^high^CD14^−^ subset in the FSC-SSC lymphocyte gate; CD56^+^ T cells and CD56^−^ T cells were defined in the CD3^+^ gate. SYTOX-Blue staining was used to detect dying cells. In CD3^+^, CD56^−^, and CD56^+^ subsets, the following cell fractions were determined: CD8^+^ and granzyme-B^+^, CD107a, naïve T cells (CCR7^+^CD45RA^+^), central memory (CM) T cells (CCR7^+^CD45RA^−^), effector memory (EM) T cells (CCR7^−^CD45RA^−^), and terminal effector memory T (TEMRA) cells (CCR7^−^CD45RA^+^). CD56^+^ cells were determined in the CD8^+^ gate. Surface expression of HLA-DR, CD16, NKG2D, PD-1, KIR2DL2/DL3, NKG2A, NKp30, CD57, NKG2C was assessed in T cells.

Most reports on COVID-19 have highlighted lymphopenia [36,37,38]. At the same time, data on the COVID-19-induced alterations in the proportions of different lymphocyte subpopulations are varying in different publications [39,40,41]. We analyzed T cells in peripheral blood mononuclear cells (PBMC) isolated from ICU COVID-19 patients (ICU), moderate severity patients (MS), convalescent plasma donors (CCP), and healthy donors who have not experienced COVID-19 (HD). Total T cells and T cell subsets were measured by flow cytometry in lymphocyte population defined as CD45^high^CD14^−^ cells. The gating strategy for determination of the lymphocytes subsets and their surface markers are presented in Figure 1.

**Figure 2:**
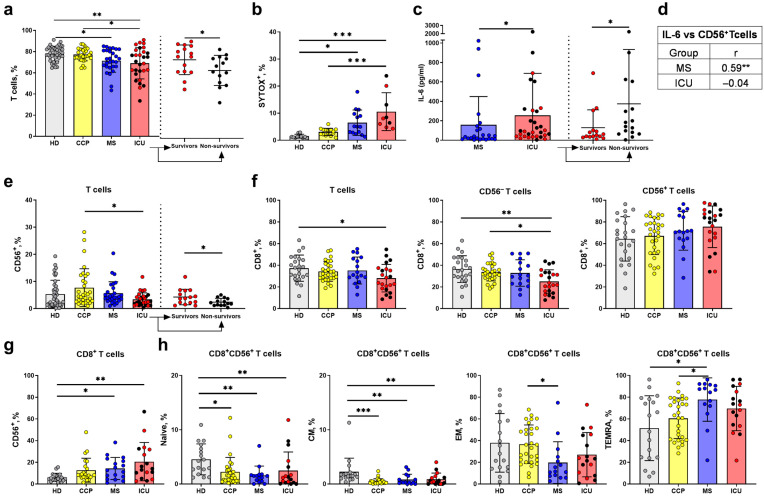
Main characteristics of the peripheral blood T lymphocytes from COVID-19 patients, convalescents, and healthy donors. (**a**) Proportion of T cells in lymphocyte population (CD14^−^ CD45^high^) measured in the following comparison groups: healthy donors (HD) (*n* = 40), COVID-19 convalescent plasma donors (CCP) (*n* = 31), moderate severity (MS) (*n* = 28) and intensive care unit patients (ICU) (*n* = 29), and comparative analysis of the survivors (*n* = 15) and non-survivors (*n* = 14) from the ICU group. (**b**) Proportion of dead cells (HD (*n* = 11), CCP (*n* = 15), MS (*n* = 15), ICU (*n* = 9)). (**c**) Concentration of IL-6 (pg/mL) measured (MS (*n* = 24) and ICU (*n* = 31)), and comparative analysis of the survivors (*n* = 15) and non-survivors (*n* = 16) from the ICU group. (**d**) Spearman correlation between IL-6 concentration (pg/mL) and the percentage of CD56^+^ T cells in the MS and ICU groups. (**e**) Percentage of CD56^+^ cells among all T cells (HD (*n* = 40), CCP (*n* = 31), MS (*n* = 28), and ICU (*n* = 29)) and comparative analysis of the survivors (*n* = 15) and non-survivors (*n* = 14) from the ICU group. (**f**) Percentage of CD8^+^ cells among T cells in the HD (*n* = 29), CCP (*n* = 29), MS (*n* = 22), ICU (*n* = 20) groups. (**g**) Percentage of CD56^+^ cells among CD8^+^ T cells (HD (*n* = 20), CCP (*n* = 22), MS (*n* = 17), ICU (*n* = 17)). (**h**) Percentage of naïve, CM, EM, TEMRA cells among CD8^+^CD56^+^ T cells (HD (*n* = 17), CCP (*n* = 29), MS (*n* = 15), ICU (*n* = 17)). Red dots stand for the survivors, black—for the non-survivors. Significance of differences in the T cell percentages (**a**) between the comparison groups and between survivors and non-survivors was determined by one-way ANOVA with post hoc Tukey’s multiple comparisons test and by unpaired *t*-test, respectively. Differences in IL-6 concentrations (**c**) and the percentages of CD56^+^ cells between survivors and non-survivors (**e**) were analyzed by the Mann–Whitney test. Significance of data differences in (**b**,**e**–**h**) was determined by the Kruskal–Wallis test followed by Dunn’s multiple comparison test. Data are presented as mean (±SD). * *p* < 0.05, ** *p* < 0.01, *** *p* < 0.001.

There was a statistically significant decrease in the proportion of T cells (CD3^+^) in the ICU and MS groups; moreover, the percentage of CD3^+^ cells was lower in the non-survivors from the ICU group (Figure 2a). Next, the viability of T cells was assessed using cytometric analysis of PBMC with the SYTOX-Blue dead cell stain. An increase in the percentage of SYTOX-Blue^+^ T cells in the ICU group was found, compared to moderate patients, convalescents, and healthy donors (Figure 2b), indicating a decrease in the viability of these cells during COVID-19.

Serum levels of IL-6, a pro-inflammatory cytokine associated with faster disease progression and risk of complications in COVID-19, were assessed [42]. Our data confirmed the elevated serum IL-6 levels in ICU patients, compared with moderate patients. Furthermore, the non-survivors had higher levels of IL-6 than the survivors in the ICU group (Figure 2c).

Recently, we have described the inflammatory context which may directly influence blood cell numbers and functions by assessing cytokine levels in serum samples from patients with moderate COVID-19 compared to healthy controls [43]. In this work, in agreement with the previously published data [44,45,46], we registered an upregulation of the “cytokine storm” markers, as well as cytokines which contribute to lymphocyte activation and exhaustion, in serum samples from COVID-19 patients. Convalescent plasma treatment was associated with the reduction in the mediators involved in T cell and neutrophil/macrophage response. In particular, infusion of plasma from COVID-19-recovered individuals resulted in a significant drop in cytokines involved in the antiviral T cell response (Figure S1a), and a decrease in the myeloid cells activation and recruitment factors (Figure S1b). In addition, IL-10, one of the major “cytokine storm” inflammatory mediators, was significantly downregulated in patients receiving convalescent plasma as compared to the control group (Figure S1c).

The T cell population was analyzed as two subsets according to the expression of CD56 marker: less differentiated CD3^+^CD56^−^ and more differentiated CD3^+^CD56^+^ T cells (Figure 1). Although a positive correlation was found between the IL-6 level and the proportion of CD56^+^ cells in the MS group (Figure 2d), we did not notice any difference in the CD56^+^ T cell content between healthy donors and COVID-19 patients. Instead, the proportion of CD56^+^ T cells was increased in the CCP group compared to the ICU group (Figure 2e). When dividing the ICU group according to the outcome of the disease, the percentage of CD56^+^ T cells was lower in non-survivors (Figure 2e).

The CD8^+^ T cell subset was also reduced in the ICU group, compared to healthy donors (Figure 2c). Moreover, when analyzing the CD56^−^ and CD56^+^ T cells separately, we found a decrease in the CD8^+^ subset only among CD56^−^ T cells in the ICU group compared to HD, while no difference was found in the proportion of CD8^+^ subset among CD56^+^ T cells (Figure 2f).

As CD56^+^CD8^+^ peripheral T cells are considered the most cytotoxic [5,7], we evaluated the content of CD56^+^ cells in the CD8^+^ subset. An increase in the percentage of CD56^+^ cells in the CD8^+^ T cell fraction during COVID-19 was observed in both ICU and MS groups compared to the HD group (Figure 2g). Thus, a decrease in the content of CD8^+^ T cells most likely takes place due to the predominant death of CD56^−^ T cells. An increased content of CD56^+^ T cells in the CCP group seems to indicate de novo T cell differentiation towards NKT-like cells.

Next, we analyzed the repertoire of the CD8^+^CD56^+^ T cell population in the context of T cell differentiation. We assessed the percentage of naïve (CCR7^+^CD45RA^+^), central memory (CM, CCR7^+^ CD45RA^−^), effector memory (EM, CCR7^−^CD45RA^−^) T cells, and TEMRA (CCR7^−^CD45RA^+^) cells in this most cytotoxic subset (Figure 1). The proportions of naïve and CM T cells in the CD8^+^CD56^+^ subset decreased in patients with COVID-19 (Figure 2h). At the same time, the MS group had the smallest proportion of EM cells and the most considerable proportion of TEMRA cells in the CD8^+^CD56^+^ subset.

A high differentiation stage of T cells and, as a result, cellular senescence is associated with the expression of the CD57 marker [47,48]. A statistically significant increase in the proportion of CD57^+^ CD56^−^ T cells in the MS patients was found compared to the ICU patients. Among CD56^+^ T cells, there were no significant differences in CD57^+^ expression in the studied groups (Figure S2).

### 2.2. Granzyme B and K562-Cell-Induced Degranulation Levels Increase in CD56^+^ T Cells during COVID-19

**Figure 3:**
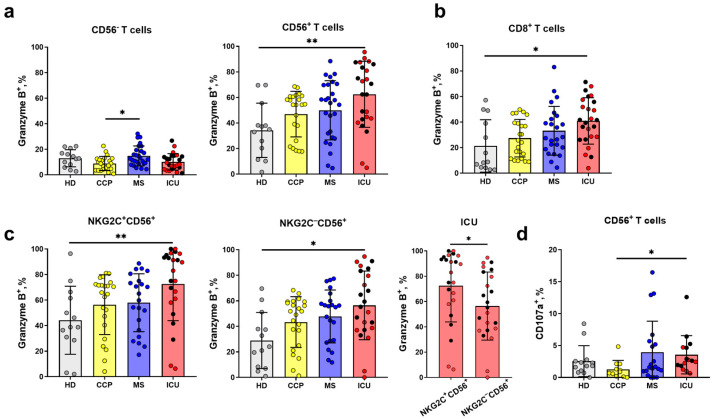
Intracellular granzyme B level in the subpopulations of T cells. (**a**) The proportion of granzyme B^+^ cells in CD56^−^ and CD56^+^ T cell subsets, measured in the following comparison groups: HD (*n* = 13) CCP (*n* = 23), MS (*n* = 27), ICU (*n* = 24). (**b**) The proportion of granzyme B^+^ cells in CD8^+^ (HD (*n* = 13), CCP (*n* = 23), MS (*n* = 22), ICU (*n* = 24)). (**c**) The proportion of granzyme B^+^ cells in NKG2C^+^CD56^+^ and NKG2C^−^CD56^+^ T cell subsets (HD (*n* = 13), CCP (*n* = 23), MS (*n* = 22), ICU (*n* = 24)). (**d**) Frequency of CD107a^+^ cells among CD56^+^ T cells (HD (*n* = 13), CCP (*n* = 11), MS (*n* = 20), ICU (*n* = 15)). Red dots stand for the survivors, black—for the non-survivors. Significance of data differences was determined by the Kruskal–Wallis test followed by Dunn’s multiple comparison test. Differences in the granzyme B levels in the T cell subsets (**c**) were determined by the Mann–Whitney test (yellow, blue, and red asterisks). Data are presented as mean (±SD). * *p* < 0.05, ** *p* < 0.01.

Intracellular production of granzyme B, the component of cytotoxic granules, in T cells is often considered as a significant parameter in the analysis of the antiviral immunity [49]. In our study, no significant changes in the intracellular granzyme B level were observed in the total T cell population during the progression of COVID-19 (Figure S3). Representative histograms of the cut-off for Granzyme B+ cells in each T cells subsets are shown in Figure S5. At the same time, the granzyme B content increased in NKT-like cell subset in the ICU group compared to the HD group, whereas the highest level of granzyme B was found in MS patients compared to ICU patients and CCP donors in the CD56^−^ T cells (Figure 3a).

Similar to the CD56^+^ T cells subset, the proportion of granzyme-B-expressing cells in the CD8^+^ T cell subset was higher in the ICU group compared to the HD group (Figure 3b).

It was shown earlier that surface NKG2C marks the most differentiated cells in both CD56^+^ and CD56^−^ T cell subsets [6]. In this study, we did not detect any statistically significant difference in the proportion of NKG2C^+^ cells among either total T cells or CD56^−^ and CD56^+^ T cell subsets between healthy donors, COVID-19 patients, and convalescents (Figure S4). At the same time, the NKG2C^−^CD56^+^ and NKG2C^+^CD56^+^ subsets in the ICU group had an increased intracellular granzyme B level compared to HDs (Figure 3c). Interestingly, NKG2C^+^CD56^+^ T cells had higher granzyme B levels compared to NKG2C^−^CD56^+^ T cells in the ICU group only (Figure 3c).

In order to evaluate NKT-like cell natural cytotoxicity, we carry out an indirect cytotoxic assay (degranulation test, based on surface CD107a detection) after 4 h co-culture with K562 cell line. NKT-like cells from patients with severe COVID-19 had higher CD107a surface level compared to recovered donors.

### 2.3. T Cells in COVID-19 Show an Activated Profile with Signs of Exhaustion

**Figure 4:**
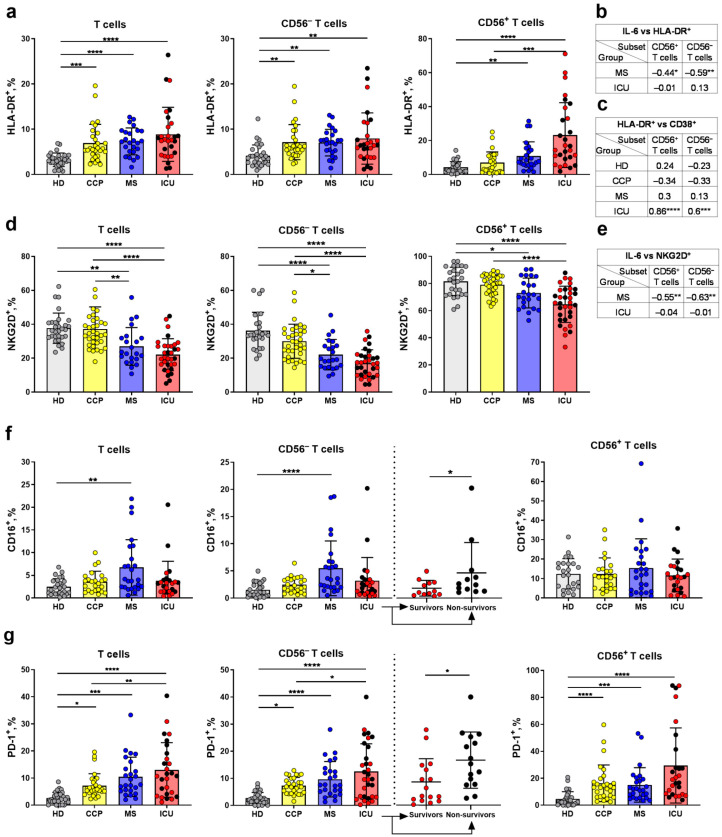
Analysis of activation and exhaustion of T cells. (**a**) The proportion of HLA-DR^+^ cells in the CD56^−^ and CD56^+^ T cell subsets, and the total T cells (HD (*n* = 28), CCP (*n* = 29), MS (*n* = 25), ICU (*n* = 26)). (**b**) Spearman correlation between the IL-6 concentration (pg/mL) and the percentage of HLA-DR^+^ cells in the CD56^−^ and CD56^+^ T cell subsets. (**c**) Spearman correlation between the percentages of HLA-DR^+^ and CD38^+^ cells in the CD56^−^ and CD56^+^ T cell subsets. (**d**) Proportions of NKG2D^+^ cells in the CD56^−^ and CD56^+^ subsets, and the total T cells (HD (*n* = 27), CCP (*n* = 35), MS (*n* = 23), ICU (*n* = 32)). (**e**) Spearman correlations between the IL-6 concentration (pg/mL) and the percentage of NKG2D^+^ cells in the CD56^−^ and CD56^+^ T cell subsets. (**f**) Proportions of CD16^+^ cells in the CD56^−^ and CD56^+^ cell subsets, and total T cells (HD (*n* = 21), CCP (*n* = 20), MS (*n* = 24), ICU (*n* = 20)). (**g**) Proportions of PD-1^+^ cells in the CD56^−^ and CD56^+^ cell subsets, and the total T cells (HD (*n* = 28), CCP (*n* = 29), MS (*n* = 25), ICU (*n* = 27)). Red dots stand for the survivors, black—for the non-survivors. Significance of data differences was determined by the Kruskal–Wallis test followed by Dunn’s multiple comparison test. Difference in the CD16^+^ (**f**) and PD1^+^ cell percentages (**g**) between survivors and non-survivors was determined by the Mann–Whitney test. Data are presented as mean (±SD). * *p* < 0.05, ** *p* < 0.01, *** *p* < 0.001, **** *p* < 0.0001.

T cell activation in COVID-19 was analyzed by the expression of HLA-DR, a subtype of the human leukocyte antigen class II, in the subpopulations of less differentiated CD56^−^ and more differentiated CD56^+^ T cells. The total T cells and CD56^−^ T cells from the ICU, MS, and CCP groups had higher proportions of HLA-DR^+^ cells compared to HD. Within the CD56^+^ T cell subset, the HLA-DR expression level was also upregulated in COVID-19 patients compared to healthy individuals, while the CCP group had a similar level to the HD (Figure 4a and Figure S6). At the same time, there were no differences in the HLA-DR expression between survivors and non-survivors in the ICU group (Figure 4a). A significant negative correlation between the expression of HLA-DR on CD56^−^ and CD56^+^ T cells and the serum level of IL-6 was observed in the MS group but not in the ICU group (Figure 4b). The CD38 molecule is induced in immune cells during the activation, but its distribution in lymphocytes is different. NK cells constitutively express CD38 molecule [50], while CD38^+^ T lymphocytes are considered activated [51]. Earlier, we have shown both CD56^−^ and CD56^+^ T cells had the highest levels of CD38 expression in the MS and the CCP groups, and decreased level in the ICU group [52]. Thus, we have additionally studied a coordination between the expression of HLA-DR and CD38 in CD56^−^ and CD56^+^ T cells subsets by the correlation analysis. Positive correlations in both CD56^+^ and CD56^−^ T cell subsets exclusively were observed in the ICU group (Figure 4c).

Activating NK cell receptors NKG2D and CD16 can be expressed on T cells at later stages of differentiation [6]. We observed a decrease in the expression of NKG2D in CD3^+^, CD56^+^ and CD56^−^ T cell subsets in patients with COVID-19 compared to HD, and this reduction was more significant in the ICU group (Figure 4d). At the same time, a negative correlation between the NKG2D expression level on CD56^−^ and CD56^+^ T cells and the IL-6 serum concentration was found in moderate patients (Figure 4e). There was an increase the CD16 expression in both total T cells and in the CD56^−^ T cell subset in the MS group compared to HD. In contrast, CD16 level in the NKT-like cell subset did not differ in all studied groups (Figure 4f and Figure S7). The non-survivors had an increased proportion of CD16^+^ cells among CD56^−^ T cells compared to the survivors (Figure 4f).

Considering that the activation of T cells after antigenic and cytokine stimulation may result in their further exhaustion [53], we evaluated the expression of the surface inhibitory receptor PD-1 as a marker of cell exhaustion [54] in T cells in the studied groups. The proportions of PD-1-expressing cells in the total T cell population and in the CD56^−^ and CD56^+^ subsets were increased in the ICU, MS, and CCP groups compared to HD (Figure 4g). However, PD-1 expression level in the CCP group was lower than in the ICU group in the total T cells and in the CD56^−^ subset (Figure 4g and Figure S7). Notably, an increased proportion of PD-1^+^ cells among CD56^−^ T cells was found in the non-survivors compared to the survived patients from the ICU group (Figure 4g).

### 2.4. The Expression Levels of NK Cell Receptors KIR2DL2/DL3, NKG2A, and NKp30 Significantly Change in T Cells in COVID-19 Patients

A range of NK cell receptors such as NKG2A and KIRs are intensively expressed by T cells at later stages of differentiation and, particularly, by NKT-like cells [6]. We have focused on investigating the expression of inhibitory NKG2A and KIR2DL2/DL3 receptors in the different T cell fractions in patients with COVID-19 and healthy donors.

**Figure 5:**
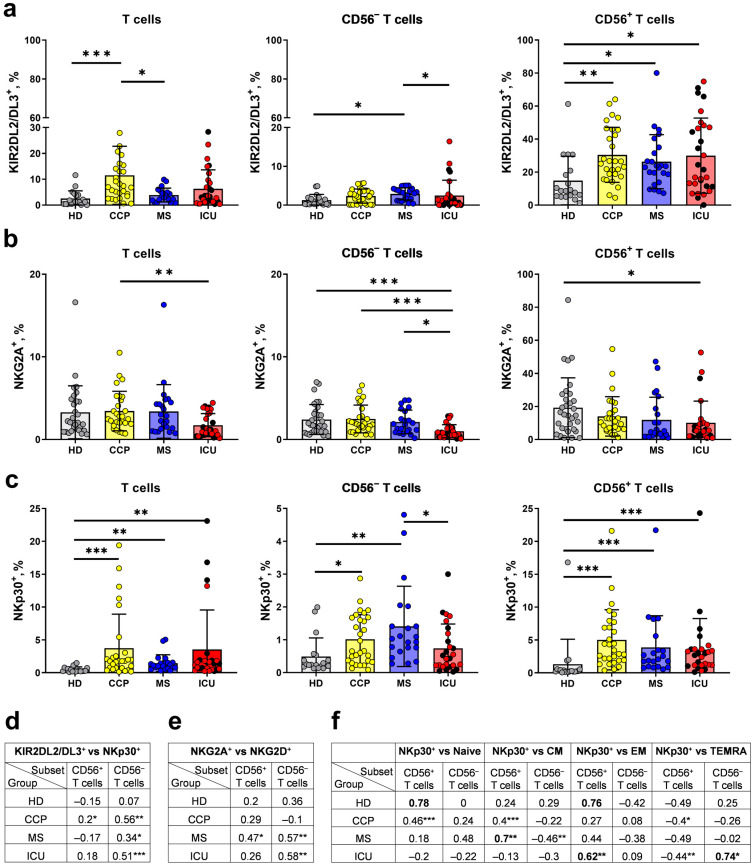
Proportions of KIR2DL2/DL3^+^, NKG2A^+^, and NKp30^+^ cells among T cell fractions. (**a**) The proportion of KIR2DL2/DL3^+^ cells in all T cells, CD56^−^ and CD56^+^ T cell subsets (HD (*n* = 19), CCP (*n* = 29), MS (*n* = 22), ICU (*n* = 31)). (**b**) The proportion of NKG2A^+^ cells in all T cells, the CD56^−^ and CD56^+^ T cell subsets (HD (*n* = 31), CCP (*n* = 29), MS (*n* = 26), ICU (*n* = 27)). (**c**) The proportion of NKp30^+^ cells in T cells, CD56^−^ and CD56^+^ T cells (HD (*n* = 19), CCP (*n* = 28), MS (*n* = 21), ICU (*n* = 26)). (**d**–**f**) Spearman correlation between the percentage of KIR2DL2/DL3^+^ and NKp30^+^ cells in the CD56^−^ and CD56^+^ T cell subsets (**d**); between the percentage of NKG2A^+^ and NKG2D^+^ cells in the CD56^−^ and CD56^+^ T cell subsets (**e**); between the percentage of NKp30^+^ and naïve, CM, EM, TEMRA cells in the CD56^−^ and CD56^+^ T cell subsets (**f**). Red dots stand for the survivors, black—for the non-survivors. Data are presented as mean (±SD). Significance of data differences was determined by the Kruskal–Wallis test followed by Dunn’s multiple comparison test. * *p* < 0.01, ** *p* < 0.01, *** *p* < 0.001.

In the whole T cell population, the highest level of KIR2DL2/DL3^+^ cells was observed in the CCP group, while in the CD56^−^ subset the proportion of KIR2DL2/DL3-expressing cells was higher in the MS group compared to the ICU and HD (Figure 5a). In the CD56^+^ subset, a statistically significant increase in the proportion of KIR2DL2/DL3^+^ T cells was detected in the ICU patients, MS patients, and convalescents compared to HD (Figure 5a). The percentage of KIR2DL2/DL3^+^ cells in the total T cells or in the CD56^+/−^ T cell subsets did not depend on the outcome of COVID-19 among the ICU group.

In the group of ICU patients, there was a significant reduction in the proportion of NKG2A^+^ cells compared to healthy volunteers in both CD56^−^ and CD56^+^ T cell subsets (Figure 5b). Similar to KIR2DL2/DL3, the proportion of NKG2A^+^ cells among the T cells did not depend on the outcome of COVID-19 (Figure 5b).

In addition to inhibitory NK cell receptors, CD8^+^ T cells can express the activating natural cytotoxicity receptor NKp30 [33]. We found an increased proportion of NKp30^+^ cells in patients with COVID-19 among total T cells and in the CD56^−^ and CD56^+^ T cell fractions compared to HD. The percentage of NKp30^+^ cells in the CD56^−^ subset was the highest in the MS group (Figure 5c). Correlation analysis revealed an association of the NKp30 and KIR2DL2/3 expression in CD56^−^ T cells in the ICU, MS, and CCP groups, but not in healthy donors (Figure 5d). Interestingly, the NKG2A^+^ cell proportions were positively correlated with NKG2D expression levels in the same subset of T cells in the MS and ICU groups (Figure 5e). An increase in NKp30^+^ cell percentage in the CD56^+^ subset in the ICU and MS groups was accompanied by an increase in the CM and EM T cell fractions, respectively (Figure 5f).

## 3. Discussion

The characteristics of lymphocytes during COVID-19 disease have been associated with lymphopenia at the late stages of the SARS-CoV-2 infection [13,21,55]. Similar to the previous studies [14,56,57], we found a decrease in the proportion of CD3^+^ T cells in the MS and ICU groups. In addition, we showed a lower percentage of CD3^+^ cells in the non-survivors from the ICU group (Figure 2a), highlighting the importance of T cells in the effective immune response to COVID-19. The decrease in CD3^+^ T cells appears to happen mainly due to the massive death of these cells: we have demonstrated that the percentage of dead cells in the ICU group was higher compared to the moderate COVID-19 patients, convalescents, and healthy donors (Figure 2b).

In early studies, both a decrease [2,22] and an increase [23] in the proportion of NKT-like cells during SARS-CoV-2 infection were reported, including a decrease in both the proportion and the absolute numbers of MAIT cells in COVID-19, while γδ T cells were reduced only in absolute numbers [58,59,60]. In our study, no decrease in the CD56^+^ T cell proportion was observed in COVID-19; nevertheless, those patients who survived after the admission to the ICU had a higher proportion of CD56^+^ cells, suggesting that this factor has a prognostic significance in the severe group (Figure 2e). In addition, an increase in the number of CD56^+^ T cells was observed in convalescents, which may be related to differentiation of T cells from CD56^−^ into CD56^+^. An upregulation of the overall pro-inflammatory cytokine levels during COVID-19 could induce such shifting in T cell subsets [43,61]. Against the background of a general decline in T cell numbers, which was shown in many works [62,63], we registered a significant decrease in the CD8^+^ T cell fraction in the ICU group, which is consistent with previous findings [64].

In concordance with a general increase in serum IL-6 level in the ICU patients observed in earlier works [58,65,66], we have noted that it was the non-survivors, who had higher levels of IL-6 (Figure 2c). Interestingly, the IL-6 level positively correlated with the proportion of CD56^+^ T cells only in the group of patients with moderate severity (r = 0.59 **, Figure 2d). This correlation supports the influence of cytokines on the number of CD56^+^ T cells. In the ICU patients, such dependence was not observed, possibly due to a massive cell loss and dysregulation of the cell composition.

CD56^+^ T cells proliferate less intensively but have an increased level of granzyme B compared to the CD56^−^ T cell subset. These cells are thought to play an essential antiviral role in the organism including through their ability to be activated in the absence of TCR-mediated stimulation (natural cytotoxicity) [31]. For example, CD56^+^ T cells are a substantial component of the cytotoxic T cell response to HCMV in healthy carriers [67]. Currently, CD8^+^CD56^+^ T cells are considered the most cytotoxic subset among all T lymphocytes [5,7]. The NK-like cytotoxic potential of these cells is mediated by a range of NK cell receptors that these cells express [5,6,31]. We found a decrease in the proportion of CD8^+^ cells in CD56^−^ but not in the CD56^+^ T cell subset in the ICU group compared to the HD, which suggests an increased survival ability of the CD56^+^CD8^+^ T cells during COVID-19 (Figure 2f,g). An increased level of resistance to apoptosis of CD56^+^ T cells has been suggested previously [31].

Terminally differentiated TEMRA cells can also acquire NK cell traits, including upregulation of CD56, to maintain a rapid effector response against tumor cells and infections in elderly people [5]. A decrease in terminally differentiated TEMRA effector cells, defined as CD45RA^+^CD62L^−^, among the CD8^+^ T cells has been shown in patients with COVID-19 compared with HD [14]. At the same time, an increase in TEMRA cell populations defined as CD45RA^+^CD27^−^CCR7^−^ was shown in the CD8^+^ T cell subset [12]. In another study, CD8^+^ TEMRA cells defined as CCR7^−^CD45RA^+^ were increased in patients with severe and critical COVID-19, but in mild patients, TEMRA CD8^+^ T cells were similar to controls [68]. In our work, the proportion of TEMRA cells defined as CCR7^−^CD45RA^+^ in all T cells and in CD8^+^ T cell subset, defined as CCR7^−^CD45RA^+^, did not differ between the patients with COVID-19 and the HD group (Figure S8). The discrepancy in the results can be explained, firstly, by a different way of determining TEMRA cells and, secondly, by differences in the studied samples of patients with COVID-19, for example, by severity. In addition, the age characteristic of the control group is significant, as the TEMRA subpopulation among T cells increases with age, particularly in the CD8^+^ compartment [69,70]. Moreover, the so-called TEMRA inflation among T cells may occur in response to a latent infection, such as HCMV [71]. However, we still detected an increase in TEMRA cells, but only in the most cytotoxic CD8^+^CD56^+^ subset in patients with moderate COVID-19 compared with healthy donors, and there was also a trend towards an increase in TEMRA cells in the ICU group (Figure 2g). On the one hand, the development of this subset may be stimulated by a high content of cytokines in the microenvironment induced by COVID-19. On the other hand, these most differentiated cells can also have an increased resistance to apoptosis [72].

The previous study showed that serum samples of the SARS-CoV-2 infected patients contained more granzyme B compared to serum samples of healthy donors [18]. T cells are one of main producers of granzyme B, and understanding which subpopulation of T cells is involved more in the cytotoxic response may reveal the reasons for the disease progression. Different studies noticed an increase in granzyme B^+^ T cell content [73]. It has been demonstrated earlier that patients with COVID-19 shifted towards a cytotoxic phenotype in the T cell compartment, as indicated by high degranulation capacity (granzyme B and perforin levels) compared to healthy controls [2]. However, another study found an abnormal CD8^+^ T cells response characterized by reduced perforin levels in patients with severe COVID-19 [1]. In our study, intracellular levels of granzyme B in CD56^+^ T cells were higher in the ICU patients compared to HD, while CD56^−^ T cells in the CCP group had reduced granzyme B^+^ cell content compared to the MS group (Figure 3a). The same phenomenon of increasing of granzyme B^+^ cells among NK cells we demonstrated earlier [43]. Thus, CD56^+^ T cells in ICU patients have a high cytotoxic potential, and CD56^+^ T cell deficiency in these patients has been shown to be associated with lethal outcome.

Hyperactivation and exhaustion of lymphocytes may contribute to the progression of COVID-19. We recorded a higher number of HLA-DR^+^ T cells in ICU patients compared to the CCP and healthy donors. An increase in HLA-DR and CD38 expression levels has been reported by us in T lymphocytes in severe and moderate cases of COVID-19 [73]. In this study, we have also observed an activated phenotype of T cells during SARS-CoV-2 infection. The total T cells and the CD56^−^ T cell fraction in the ICU, MS, and CCP groups had a higher proportion of HLA-DR^+^ cells compared to healthy controls, while in the CD56^+^ T cell fraction, COVID-19-mediated activation was registered only in convalescents (Figure 4a). Correlation analysis of the CD38 and HLA-DR expression levels revealed a positive correlation between these receptors in CD56^−^ T cells obtained from ICU and moderate severity COVID-19 patients. This fact supports a high activation level of the CD56^−^ T cells, which apparently leads to cell exhaustion and death.

The inhibitory PD-1 receptor demonstrates stable expression on the resting T cells and overexpression during activation [54,74]. In this work, we analyzed the expression level of PD-1 as an indicator of cell hyperactivation and exhaustion. During T cell activation in response to viral antigen recognition the inhibitory PD-1 receptor serves for a limitation of the protective immunity preventing an excessive response [75]. PD-1 may be also overexpressed on T cells during acute T cell activation without a decline in the cell functionality, thus, PD1 should not be considered as an exhaustion-specific marker [76]. Our data (Figure 4g), in accordance with earlier observations, prove that T cells from COVID-19 patients have significantly higher PD-1 levels compared to healthy controls [62,77]. We observed higher proportions of HLA-DR^+^ and PD-1^+^ T cells during severe and moderate COVID-19. Thus, we can consider that T cell depletion is accompanied by signs of their hyperactivation and exhaustion in COVID-19.

KIR, NKG2D, and CD16, as well as several other NK cell receptors, can upregulate at late stages of T cell differentiation [6]. For the NK cells, signal transduction from the activating receptor NKG2D is sufficient for the direct destruction of target cells, while in T cells, simultaneous activation of the T cell receptor is required for the functioning of NKG2D as a costimulatory molecule [78]. As a result of this “double” activation, cytotoxic and proliferative responses of T cells are enhanced [79]. Soluble ligands of the activating receptor NKG2D from the MIC and ULBP families were shown to be increased in moderate COVID-19 [23]. After binding to the ligand, the NKG2D receptor is internalized, and persistent binding causes a decrease in NKG2D surface expression, which leads to functional impairment of the NKG2D-expressing NK cells [80]. In this study, in patients with COVID-19 of any severity, as well as in CCP, we registered a decrease in the proportion of NKG2D^+^ cells among both less differentiated CD56^−^ and more differentiated CD56^+^ T cells compared with the HD group. The lowest percentage of NKG2D-expressing T cells was found in ICU patients (Figure 4d). Previously, we have also shown a decrease in the proportion of NKG2D^+^ NK cells in ICU and moderate patients with COVID-19 [43], that via IL-6 could reduce the activity of NK cells by suppressing the expression of NKG2D [81]. Interestingly, in CD56^−^ and CD56^+^ T cells we found a negative correlation between NKG2D expression and serum IL-6 level in moderate patients, but not in the ICU group (Figure 4c). Thus, it can be assumed that constant antigenic and cytokine (IL-6) stimulation seems to reduce the expression of the NKG2D receptor not only on NK cells but also on T cells during COVID-19.

Some effector memory T cells are known to express FcγRIIIa (CD16) and are involved in mediate antibody-dependent cellular cytotoxicity (ADCC) ex vivo [82]. Moreover, ADCC was revealed as an essential feature of the anti-SARS-CoV-2 immune response [83]. TCRαβ^+^CD8^+^ T cells in chronic hepatitis C virus have been shown to express CD16, acquiring functional properties similar to CD16^+^ NK cells [84]. The percentage of NKT-like cells with cytotoxic phenotype CD3^+^CD56^+^CD16^+^ was observed to be increased in patients with critical COVID-19, while the proportion of these cells expressing granzyme B was decreased in ICU patients along with a decrease in their cytotoxicity [68]. Highly activated cytotoxic CD16^+^ T cells developing in COVID-19 have been shown recently to contribute to microvascular endothelial cell injury and release of mediators which attract neutrophils and monocytes during COVID-19 [85]. In our previous work, CD16 expression on NK cells was shown to be decreased in ICU and moderate patients with COVID-19 [43], while in this work, no significant differences in the proportion of CD16^+^ NKT-like cells were recorded between the COVID-19 patient groups. Interestingly, the proportion of CD16^+^ in the CD56^−^ T subset in samples obtained from MS patients was higher compared to the HD group (Figure 4f), which may serve as a compensatory mechanism in response to a decrease in this receptor on NK cells. Infusion of plasma from the COVID-19-recovered individuals resulted in a significant drop in cytokine levels (Figure S1a), and in a decrease in the levels of myeloid cells activation and recruitment factors (Figure S1b). Given the fact that highly activated cytotoxic CD16^+^ T cells, capable of immune complex-mediated, TCR-independent cytotoxicity, are recruited in severe COVID-19 [85], the selection of potential donors with high levels of plasma virus-neutralizing activity is especially important [86].

CD94/NKG2A heterodimer is an inhibitory receptor that confers the ability of NK cells to discriminate between self and non-self. In an early work on COVID-19, increased expression of NKG2A on CD8^+^ T cells and NK cells was registered and considered as a signal of depletion and inhibition of the antiviral immune response [32], which could be associated with the progression of COVID-19 in hypertensive patients [87]. The abolition of NKG2A^+^ NK cell inhibition by the viral peptide SARS-CoV-2-Nsp13 in patients with COVID-19 leads to their activation through the HLA-E [88]. We have not found any increase in NKG2A expression in T cells during COVID-19 in our work. On the contrary, in the ICU group, both in the total CD3^+^ and in the CD56^−^ and CD56^+^ T cell subsets, we recorded a reduced expression of the NKG2A receptor compared to the HD group (Figure 5a). Downregulation of NKG2A may contribute to the pathological T cell activation in patients with severe COVID-19.

Peripheral blood cells include a small subset of effector memory T cells expressing KIR receptors [89]. Most KIR^+^ T cells also co-express CD56 molecule [7,31]. These cells are substantial in the struggle with infections such as HCMV and hepatitis C virus [84,90,91]. A unique subset of KIR^+^CD56^+^ T cells has been found to contribute to the T cell response to the HCMV reactivation [31]. In this study, the proportion of KIR2DL2/DL3^+^ cells among CD56^+^ T cells was higher in the ICU patients and convalescents compared to healthy donors (Figure 5a). It is possible that in the ICU patients with severe COVID-19, T cells rapidly differentiate from CD56^−^KIR2DL2/DL3^+^ to CD56^+^KIR2DL2/DL3^+^. In convalescents, the proportion of CD56^−^KIR2DL2/DL3^+^ T cells was significantly higher than in the ICU patients and healthy donors (Figure 5a), indicating acquisition of KIR2DL2/DL3 expression in T cells.

A small population of CD8^+^ T cells expressing NKp30 in the peripheral blood of healthy donors is identified as innate-like CD8^+^ T cells. Innate-like CD8^+^ T cells expressing NKp30 can differentiate from a population of peripheral blood NKp30^−^CD8^+^ T cells under IL-15 stimulation [33]. In our previous study, we did not notice any differences between severe patients and healthy donors in terms of NKp30 expression by NK cells against the background of elevated serum IL-15 levels [43]. In this study, increased proportions of NKp30^+^ cells among all T cells and in the fractions of CD56^−^ and CD56^+^ T cells were found in patients with COVID-19 of any severity and in convalescents. Thus, we have shown that against the background of COVID-19, under which the concentration of IL-15 increases, T cells acquire the features of innate immune cells.

## 4. Materials and Methods

### 4.1. Characteristics of COVID-19 Patients and Donors

The blood samples from patients and healthy donors were obtained at the Federal Research and Clinical Center for Specialized Types of Medical Care and Medical Technologies of the Federal Medical and Biological Agency (FMBA). Written informed consent of each participant was received.

The groups of patients were formed in accordance with the inclusion criteria: the confirmed presence of SARS-CoV-2 infection by PCR examination of the upper respiratory tract secretions, by presence of a clinical and radiological symptoms (characteristic signs of polysegmental viral pneumonia, corresponding to the classification of CT2-CT3-CT4 COVID-19). Saturation level SpO2 ≤ 93%, intermittent mandatory ventilation (IMV) supporting, treatment in the pulmonology department of FMBA, the absence or the presence of diabetes mellitus, obesity, cardiovascular disease CVD, and other comorbid conditions that worsen the prognosis for recovery did not prevent patients from being included in the study. The exclusion criteria: presence of a blood disease or an oncological disease.

According to the severity of the COVID-19 two groups of patients were defined: (1) 34 COVID-19 patients of the intensive care unit (ICU patients) (aged from 30 to 82 years, median age 61 years; SD +/− 11,5; 60% male); (2) 28 patients with moderate COVID-19 severity (MS) (aged from 23 to 93 years, median age 59 years; SD +/− 17,5; 64% male). The healthy donors were separated into two groups: (1) 33 COVID-19 convalescent plasma donors (CCP), recovered from COVID-19 less than a month ago, with a high SARS-CoV-2-specific antibody titer in plasma (aged from 33 to 54 years, median age 45 years; SD +/− 6,8; 60% male); (2) 48 volunteers (HD), who had no signs of COVID-19 infection in the last 6 months (aged from 28 to 73 years, median age 40 years; SD +/− 14,3; 41% male).

### 4.2. Peripheral Blood Mononuclear Cell Isolation

The blood samples from EDTA-containing test tubes were diluted in PBS solution (PanEco, Russian Federation) in 1:1 and centrifuged in a ficoll gradient with a density of 1.077 g/cm^3^. Separated peripheral blood mononuclear cells (PBMC) were immediately used in phenotype and viability analysis, functional test by flow cytometry; plasma was stored at −60 °C until used.

### 4.3. Phenotype and Viability Analysis

PBMC samples were stained with the panels of fluorochrome-conjugated monoclonal antibodies, which are in the Supplementary Table S1. The list of mouse anti-human antibodies included: NKG2C-FITC (4:100, clone REA205), CD3-Vioblue (0.4:100, clone BW264/56), CD56-APC-Vio770 (clone REA196), CD45-VioBlue (2:100, clone REA747), CD14-PE-Vio-770 (0.4:100, clone TÜK4), CD16-APC (3:100, clone REA423) from Miltenyi Biotec, Bergisch Gladbach, Germany; HLA-DR-PE (3:100, clone L243, Sony Biotechnology, San Jose, CA, USA), CD3-PerCP (5:100, clone HIT3a), CD3-FITC (3:100, clone FIT3a), CD45RA-APC (2:100, clone HI100), CD197-FITC (5:100, clone G043H7), KIR2DL2/L3- APC (1:100, clone DX27), NKp30-PE (3:100, clone P30-15), NKG2D-PE (3:100, clone 1D11), CD45-PerCP (2:100, clone 2D1), CD57-APC (4:100, clone HNK-1) from Sony Biotechnology, San Jose, CA, USA; CD56-APC (3:100, clone N901) from Beckman Coulter, Brea, CA, USA, PD-1-Alexa Fluor 647 (1:100, clone EH12.2H7) from BioLegend, San Diego, CA, USA.

Viability staining with SytoxBlue Dead Cell stain was performed (Invitrogen, Waltham, MA, USA). The gating strategy is presented in Figure 1.

### 4.4. Flow Cytometry and Data Analysis

Samples were analyzed using a MACSQuant 10 flow cytometer (Miltenyi Biotec, Bergisch Gladbach, Germany) equipped with lasers λ = 405 nm, λ = 488 nm, λ = 635 nm; threshold was set to cut off events with low CD45 staining.

Data processing was conducted by FlowJo X 10.0.7r2 (FlowJo LLC, Ashland, OR, USA).

### 4.5. Intracellular Staining of Granzyme B

Intracellular staining with Alexa Fluor 647—labeled antibodies to granzyme B (0.1:100, clone GB11, BioLegent, San Diego, CA, USA) in preliminary fixed PBMC samples (MInside Stain Kit, Miltenyi Biotec, Bergisch Gladbach, Germany) was performed, after that samples were analyzed by flow cytometry.

### 4.6. Functional Test: Degranulation

Flow cytometry was employed to analyze the natural cytotoxic ability of NKT-like cells in PBMCs by determining their degranulation level. The PBMCs were stimulated with IL-2, 500 U/mL, for an overnight period (Sci-Store, Moscow, Russia). NKT-like cells degranulation was detected by the level of expression of the lysosomal marker LAMP-1 (CD107a) using CD107a-VioBlue monoclonal antibody (0.5:100, clone REA792, Miltenyi Biotec, Bergisch Gladbach, Germany), as described earlier [43]. Cells were labeled with fluorescent-labeled monoclonal antibodies to CD14, CD45, CD56, CD3, and NKG2C, and analyzed by flow cytometry. NKT-like cells were identified as CD3^+^CD56^+^ cells.

### 4.7. Assessment of Cytokine Production

Unbiased analysis of serum cytokines was performed using Luminex xMAP multiplex technology and the MILLIPLEX MAP Human Cytokine/Chemokine Magnetic Bead Panel Kit, according to the manufacturer’s standard protocol (Merck, Rahway, NJ, USA). Data processing was carried out using Belysa software v1.1.0 (Merck, Rahway, NJ, USA) at the Resource Center “Cell Technology and Immunology”, Sirius University of Science and Technology.

### 4.8. Statistical Analysis

GraphPad Prism 8.00 software (StatSoft Inc., Tulsa, OK, USA) and presented as mean ± standard deviation. The ANOVA test for parametric data was carried out using post hoc Tukey’s multiple comparisons test; Kruskal–Wallis test followed by Dunn’s multiple comparison test for nonparametric samples was used. For comparison analysis of two groups, *t*-tests for parametric data and the Mann–Whitney U test for nonparametric data were performed. Pearson’s correlation test for data with normal distribution and Spearman’s correlation test for abnormally distributed data was applied for correlation analysis.

## 5. Conclusions

In this work we have studied the COVID-19-induced redistribution of T cell subsets with a special focus on CD56^−^ and CD56^+^ (NKT-like cells) fractions. The NKT-like cell subset was decreased in patients with lethal outcome of COVID-19, which implies an antiviral role of this cell subpopulation and emphasizes its prognostic value. Along with an increase in the expression levels of HLA-DR and PD-1, and elevated IL-6 serum concentration, an increase in the CD16^+^ cell proportion among CD56^−^ T cells may be considered the factor associated with increased COVID-19-mediated mortality. Moreover, a decrease in the expression of the NKG2D and NKG2A NK cell receptors observed in T cells of COVID-19 patients can potentially serve as prognostic indicators of the disease severity. Along with the depletion and hyperactivation of T cells, an elevated granzyme B level and a tendency to an increased TCR-independent functional activity were observed in CD56^+^ cells of patients with COVID-19. We have shown that CD56^+^ T cells increase expression of the KIR2DL2/DL3 and NKp30 receptors during COVID-19. The elevated KIR2DL2/DL3 and NKp30 levels were maintained in newly generated CD56^+^ T cells after the disease recovery. KIR^+^CD8^+^ T cells were previously supposed to play a regulatory role in several infection and autoimmune diseases [30]. Comprehensive analysis of the various fractions, which compose the heterogeneous CD56^+^ T cell subset, may promote significant progress in the study of SARS-CoV-2 and other viral infections. Single cell analysis of T cells, focused on NK cell receptors and signaling molecules, such as KIRs, NKp30, NKG2C, CD16, NKG2D, and FcεRIγ, may provide valuable insights into the role of such “innate-like” T cell subpopulation in the development of COVID-19 and other viral infections.

## Data Availability

Data are available on request.

## Supplementary Materials

The following supporting information can be downloaded at: https://www.mdpi.com/article/10.3390/ijms24109047/s1.
