# Supplementary material for: Alterations in the CD56− and CD56+ T Cell Subsets during COVID-19
Source: Int J Mol Sci. 2023 May 20;24(10):9047. doi: 10.3390/ijms24109047 (PMC10219320; doi:10.3390/ijms24109047)
Supplement: Supplementary file 1 [file ijms-24-09047-s001.zip › ijms-2402206-supplementary.pdf]

Supplement

Supplementary Table S1. The panels of fluorochrome-conjugated monoclonal antibodies were used in the investigation.

	Panel 1	Panel 2	Panel 3	Panel 4	Panel 5	Panel 6
Vio-blue	CD3	CD3	Sytox	CD3	CD45	CD45RA
FITC	NKG2C	-	CD3	CD38	NKG2C	CCR7
PE	NKG2A	NKp30	NKG2D	HLA-DR	CD8	CD8
Per-Cp	CD45	CD45	CD45	CD45	CD3	CD45
PE-Cy7	CD14	CD14	CD14	CD14	CD14	CD14
APC	CD57	KIR2DL2/DL3	CD16	PD-1	Granzyme B	CD3
APC-Cy7	CD56	CD56	CD56	CD56	CD56	CD56

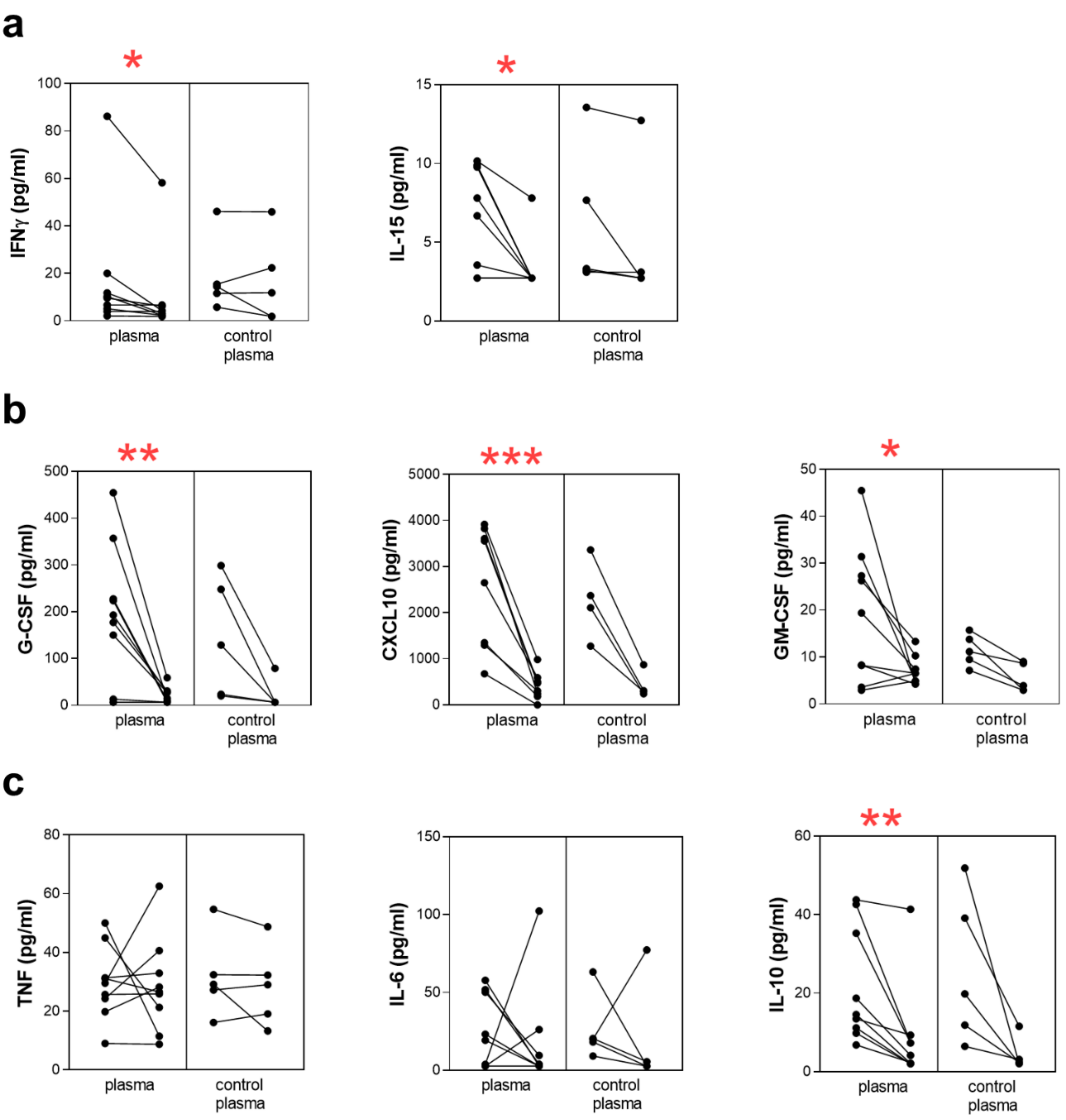

Supplementary Figure S1. Assessment of the cytokine and chemokine concentrations in the serum of patients before and 2-3 days after the convalescent plasma or control plasma infusion. Concentration of (a) IFNγ and IL-15, (b) G-CSF, CXCL10 and GM-CSF and (c) TNF, IL-6 and IL-10 in pg/mL in the serum. Data are presented as individual values before (left dots) and after (right dots) the infusion, dots from the same patient are linked. *p<0.05, **p<0.01, ***p< 0.001, ****p<0.0001.

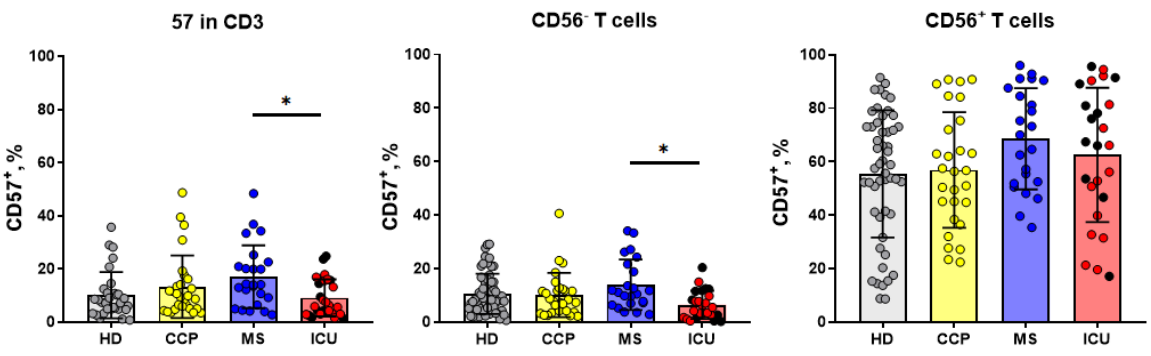

Supplementary Figure S2. The proportion of CD57$^+$ cells among T cells, CD56$^-$ and CD56$^+$ T cells measured in the following comparison groups: HD (n=62), CCP (n=29) MS (n=26) ICU (n=27). Data are presented as the mean (± SD). *p<0.01.

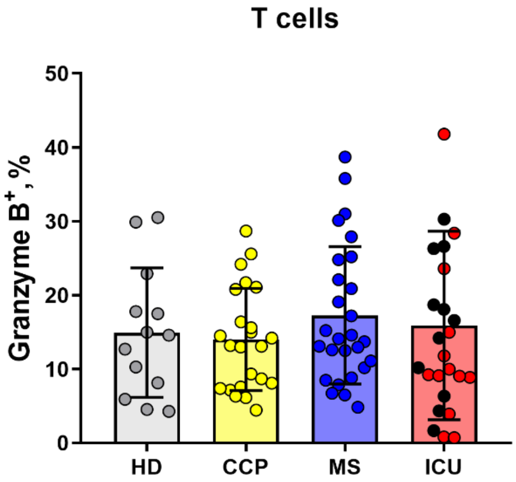

Supplementary Figure S3. Intracellular granzyme B level in all T cells.

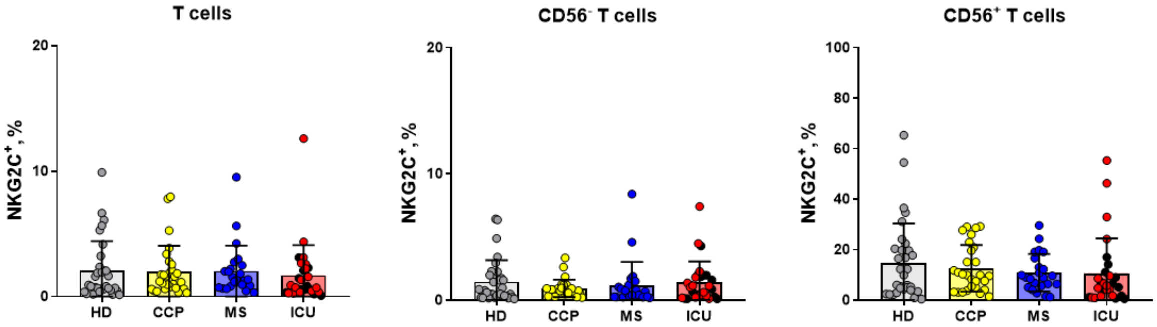

Supplementary Figure S4. The proportion of NKG2C$^+$ cells among T cells, CD56- and CD56+ T cells measured in the following comparison groups: HD (n=31), CCP (n=29) MS (n=26) ICU (n=27). Data are presented as the mean (± SD).

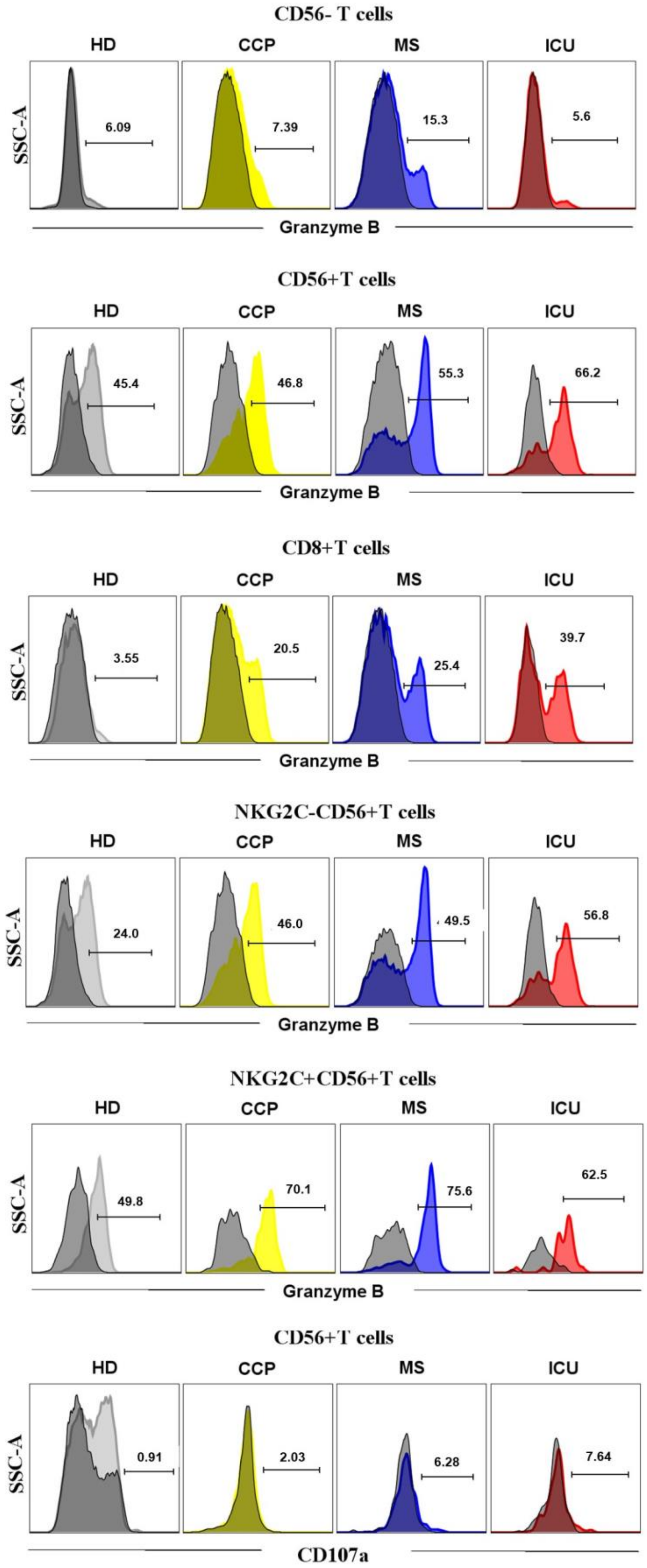

Supplementary Figure S5. Representative histograms of the cut-off for Granzyme B+ cells in each T cells subsets and CD107a in CD56+ T cells in all studied groups with the respective controls without target cells (K562) or FMO (fluorescence minus one) controls (gray).

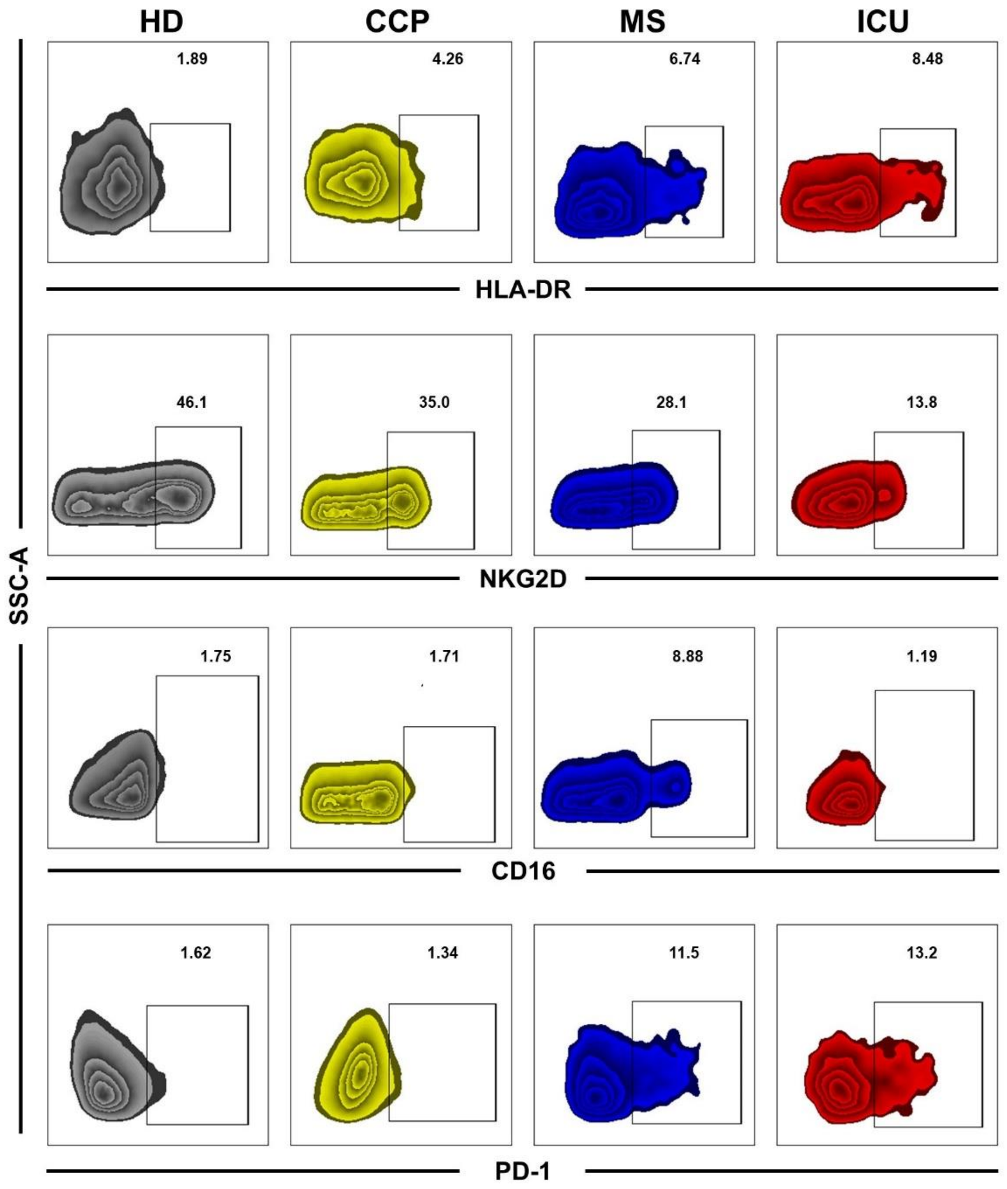

Supplementary Figure S6. Representative dot plots of surface expression of HLA-DR, NKG2D, CD16 and PD-1 were analyzed in T cells by flow cytometry after staining with fluorescent-labeled specific monoclonal antibodies.

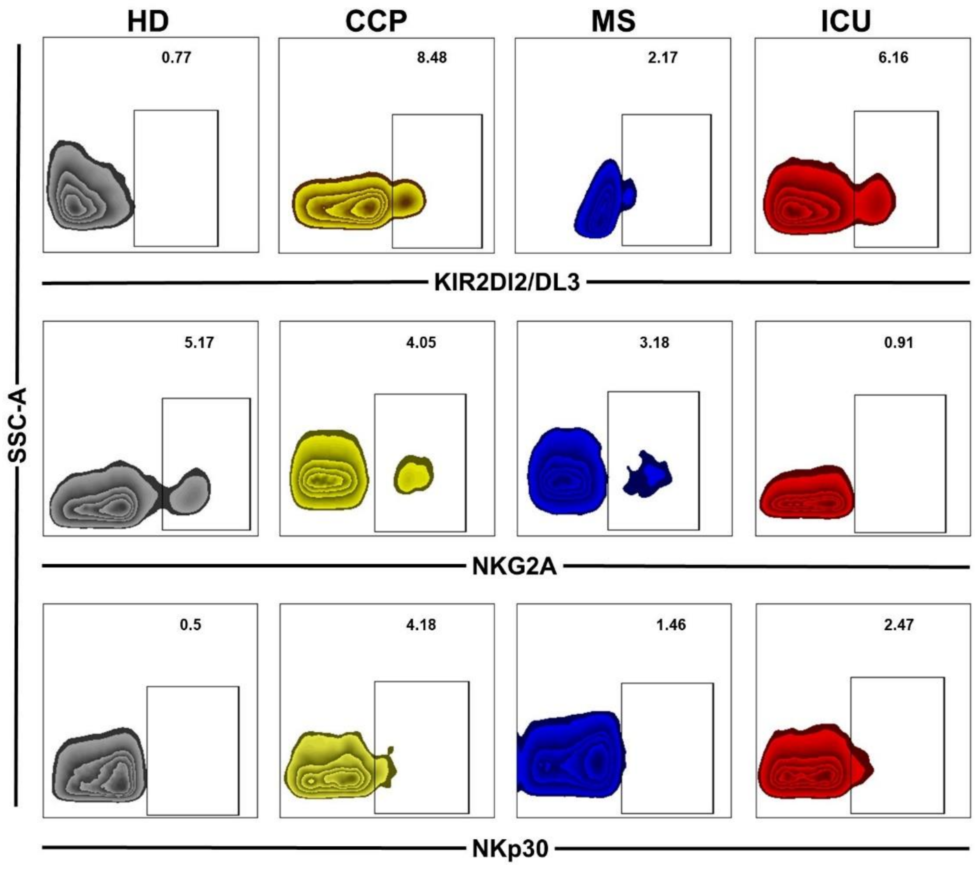

Supplementary Figure S7. Representative dot plots of surface expression of NKG2A, KIR2DL2/DL3 and NKp30 were analyzed in T cells by flow cytometry after staining with fluorescent-labeled specific monoclonal antibodies.

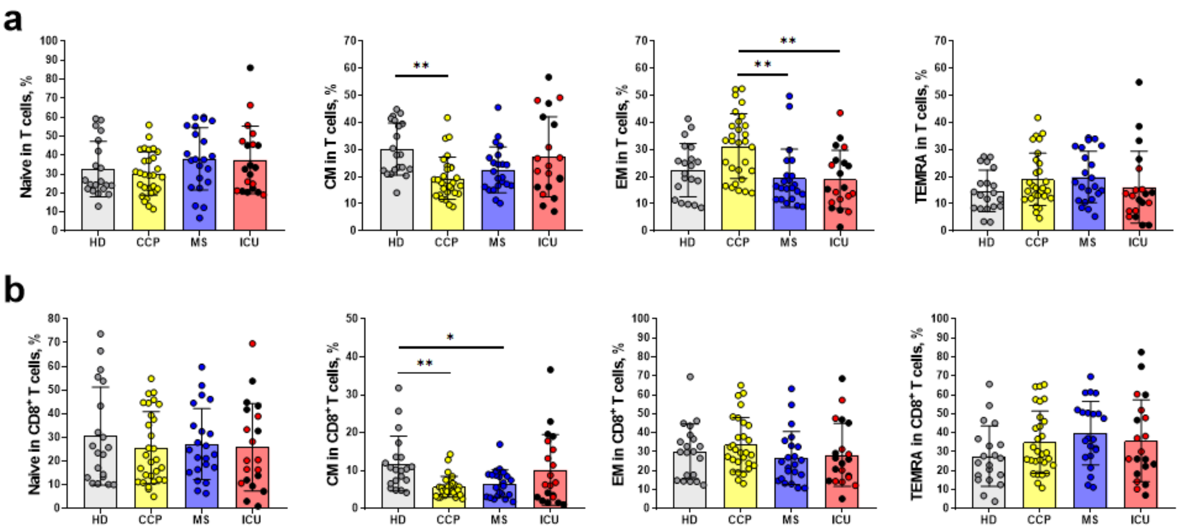

Supplementary Figure S8. Percentage of naive, CM, EM, TEMRA cells (a) among T cells in HD (n=17), CCP (n=29), MS (n=15), ICU (n=17) groups (b) among CD8+ T cells in HD (n=17), CCP (n=29), MS (n=15), ICU (n=17) groups. Data are presented as the mean (± SD). *p<0.05,**p<0.01.
